# Comparison of Nutrition Risk Screening 2002 and Subjective Global Assessment Form as Short Nutrition Assessment Tools in Older Hospitalized Adults

**DOI:** 10.3390/nu13010225

**Published:** 2021-01-14

**Authors:** Łukasz Kroc, Elizaveta Fife, Edyta Piechocka-Wochniak, Bartłomiej Sołtysik, Tomasz Kostka

**Affiliations:** Healthy Ageing Research Centre (HARC), Department of Geriatrics, Medical University of Lodz, Pieniny 30, 92-003 Lodz, Poland; lukasz.kroc@umed.lodz.pl (Ł.K.); elizaveta.fife@umed.lodz.pl (E.F.); edyta.piechocka-wochniak@umed.lodz.pl (E.P.-W.); bartlomiej.soltysik@umed.lodz.pl (B.S.)

**Keywords:** malnutrition, NRS-2002, SGA, VES-13, Comprehensive Geriatric Assessment

## Abstract

The aim of the present study was to compare two widely recommended short nutrition assessment tools—Nutrition Risk Screening 2002 (NRS-2002) and Subjective Global Assessment Form (SGA)—with other Comprehensive Geriatric Assessment (CGA) measurements. The study included 622 consecutively hospitalized older subjects, aged 81.7 ± 7.8 years. The criteria to participate were the ability to communicate and given consent. Both NRS-2002 and SGA were inversely related to anthropometric measurements, functional assessment tests, Mini-Mental State Examination (MMSE) and positively associated with the Vulnerable Elders Survey-13 (VES-13) score. Results of SGA and NRS-2002 were not related to sex and 15-item Geriatric Depression Scale (GDS) score. Comparison of well-nourished subjects and patients with suggested problems with nutrition according to NRS-2002 (0–2 vs. 3–7) and SGA (A vs. B + C) gave comparable results. Both nutritional scales at given cut-off points similarly discriminated anthropometric data and other CGA tools in the populations of well-nourished vs. malnourished hospitalized older subjects. In conclusion, we can recommend using both NRS-2002 and SGA to detect malnutrition or risk of malnutrition in a routine clinical practice of the geriatric department ward.

## 1. Introduction

Malnutrition (undernutrition) is one of the most common problems in aging societies. In Europe, an estimated 33 million people are at risk of malnutrition [1]. Studies show that up to one third of patients in hospitals and nursing homes are at risk of undernutrition, as are 10% of individuals over the age of 65 in the European Union (EU) [2,3]. Malnutrition is associated with impaired muscle function, decreased bone mass, immune dysfunction, reduced cognitive functioning, anemia, prolonged hospitalization, and increased risk of frailty, falls, morbidity and mortality [4,5].

The prevalence of malnutrition is even higher in geriatric hospitalized population—between 30% and 60% [6,7,8,9]. Therefore, valid and quick detection of malnutrition is of utmost importance in hospitalized elderly and several short nutritional tests have been proposed to check for malnutrition in that population. Nutrition Risk Screening 2002 (NRS-2002) [10,11] and Subjective Global Assessment Form (SGA) [12] are among the most widely used [13].

Although both tools have become commonly used in hospitalized patients in various clinical settings, current literature shows a relatively small amount of data about the validation of NRS-2002 and SGA scales in hospitalized older patients and its relationship with other widely used geriatric measures–especially in large population studies. In older adults with multiple deficiencies and comorbidities, the routine format of medical examination and other common tests and procedures is usually not sufficient. Therefore, the Comprehensive Geriatric Assessment (CGA) has been developed to address patients’ problems with medical comorbidities, functional status and psychosocial capacities [14]. The aim of this study was to assess concurrent validity and compare NRS-2002 and SGA with other tools commonly used in the CGA in a large population of hospitalized older subjects.

## 2. Materials and Methods

### 2.1. Design of the Study and Participants

**Figure 1:**
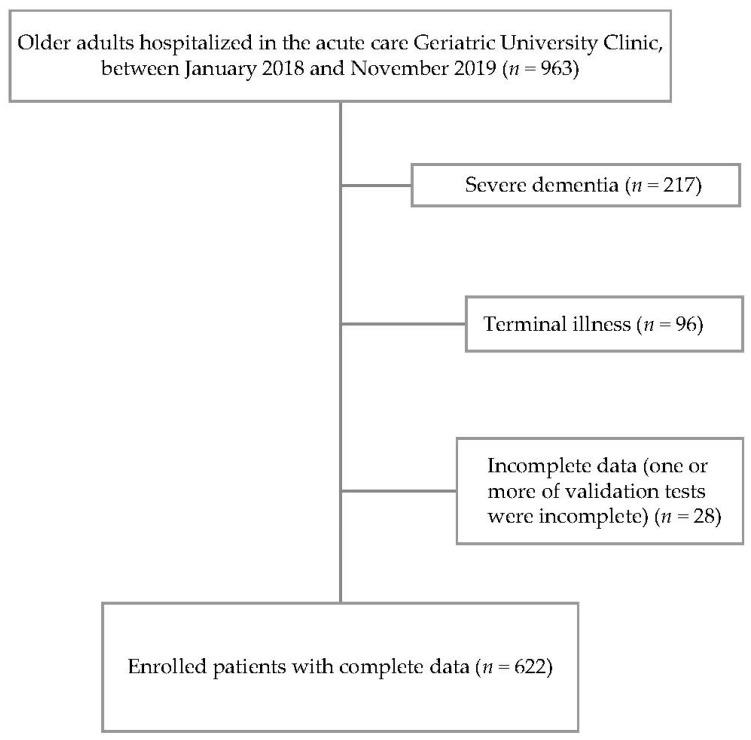
Flow chart of enrollment for the study.

The study initially included 963 older people, aged 60 and above years old, who were hospitalized in the acute care Geriatric University Clinic, Central Veterans’ Hospital in Lodz (Poland), between January 2018 and November 2019. The criteria for the participation in this study were efficient verbal communication and given consent. Out of the 963 hospitalized patients, 341 were further excluded due to incomplete data (one or more of validation tests were incomplete), severe dementia or terminal illness. Therefore, 622 patients with completed data were finally included to the study (Figure 1). The following tests were conducted in all subjects: the NRS-2002 and SGA to measure nutritional status, Activities of Daily Living (ADL) [15] and Instrumental Activities of Daily Living (IADL) [16] to measure functional status, Mini Mental State Examination (MMSE) [17] to measure cognitive status, Geriatric Depression Scale (GDS) [18] to measure depression status and Vulnerable Elders Survey -13 (VES-13) [19] was used as a screening tool for frailty. All the tests were conducted by the physicians of the geriatric ward at admission.

### 2.2. Nutritional Questionnaires

NRS-2002 was designed as a tool to identify patients at nutritional risk [10,11]. Nutritional risk was assessed through two criteria: impaired nutritional status and disease severity. A score between 0 and 3 was given for each criterion. Nutritional status was determined by three variables: BMI, recent body mass loss, and food intake during the week before hospital admission. Disease severity was analyzed by assessing increased nutritional requirements caused by recent medical history (falls, fractures, operations, oncologic and intensive care therapy) and concomitant chronic diseases. For people aged 70 and above years old, an additional extra point was added. The NRS-2002 score is a sum of the total of the nutritional score, severity of disease score and the age adjustment score. The total number of points ranges from 0 to 7. Patients with a score of 3 and more are suggested to have problems with nutrition [20].

SGA is determined on the basis of medical history about changes in nutrients intake, body mass loss, symptoms affecting oral intake (diarrhea, vomiting, nausea, dysphagia, oral problems), functional capacity (fatigue and progressive loss of function), and on physical examination findings such as subcutaneous fat, muscle wasting, presence of edema and ascites. Patients with severe malnutrition were classified as C (or 3 points), moderate malnutrition as B (or 2 points), and normal nutrition as A (or 1 point). The information necessary to fulfill the SGA was collected directly from the patients, or if this was not possible, the data were provided by accompanying family members [12].

### 2.3. Other Tools

ADL scale (Katz scale) evaluates such parameters as for example, the ability to maintain hygiene or to feed him/herself. Low scores on this scale indicate an inability to function independently. Patients score 1 point for positive responses of the type: “I do not have any problem with this ability”. The total number of points ranges from 0 to 6, with scores of 5 and 6 indicating patients in good condition [15].

IADL scale (Lawton scale) examines the ability of seniors to manage their life in the modern environment. The IADL takes into account for example, the ability to use the phone or managing money. Patients receive 1 point for positive responses indicating “I do not have any problem with this ability”. The total number of points ranges from 0 to 8, with scores of 7 and 8 indicating good condition [16].

MMSE is the most commonly used test for problems with memory or other mental abilities. It can be used to help diagnose dementia. This test consist of questions about orientation concerning time and place, attention and calculation, recall, language and praxis. The maximum possible score is 30 points, with a score of 24 points or more indicating that patients do not have problems with memory loss [17].

GDS has 15 questions describing the well-being of the patient. The maximum possible is 15, with scores of 5 or less indicating no problems with depression [18].

VES-13 includes questions about age (<75 years = 0 points, 75–84 years = 1 point, age ≥ 85 years = 3 points), self-rated health status (poor or fair = 1 point, good or average = 0 points) and two main sections: one about physical functioning and the other about the need for assistance with daily activities. The whole VES-13 consists of 13 questions, with a maximum score of 10 points for the worst prognosis [19].

### 2.4. Statistical Analysis

Data was verified for normality of distribution (Kolmogorov-Smirnov test) and equality of variances (Levene’s test). Pearson’s and Spearman’s correlation coefficients were used to measure the strength and direction of the relationship between two variables. Values of NRS-2002 and SGA were further dichotomized to compare well-nourished subjects with patients suspected of malnutrition (NRS-2002 0-2 vs. NRS-2002 3-7 and SGA A vs. SGA B + C). The sensitivity (the proportion of SGA B + C cases correctly identified as NRS-2002 3-7 cases) and specificity (the proportion of SGA A correctly identified as NRS-2002 0-2 cases) of NRS-2002 to detect malnutrition as compared to SGA was calculated. The one-way analysis of variance (ANOVA), Mann-Whitney test and chi-square test (with Yates’ correction for 2 × 2 tables) were used to test for differences between the sex and nutritional status groups. Statistical analysis was carried out using Statistica 13.1 software (StatSoft Polska, Cracow, Poland). The quantitative data were expressed as mean ± standard deviation. The limit of significance was set at *p* = 0.05.

### 2.5. Ethical Certification

The study was approved by the Ethics Committee of the Medical University of Lodz (approval number: RNN/300/17/KE) and written informed consent was obtained from all subjects.

## 3. Results

Patient characteristics is presented in Table 1. The reasons for hospitalization were very diverse, ranging from anemia and pneumonia, to gastrointestinal bleeding, loss of body mass, diagnosis of physical or cognitive function decline, stroke or diabetes mellitus. The majority of patients had several concomitant diseases. Mean age of the whole population was 81.7 ± 7.8 years. Women had lower body mass, all the circumferences, ADL and IADL, and higher GDS and VES-13 than men. NRS-2002 and SGA were virtually the same in women and men.

Table 2 and Table 3 show distribution of scores of the two nutritional scales–NRS-2002 and SGA. The majority of the population was not malnourished according to both scales. The distribution of both NRS-2002 and SGA was very similar in women and men.

Table 4 shows Spearman correlation coefficients of NRS-2002 (0–7 points) and SGA (1–3 points) with age, anthropometric data and other CGA tools. NRS-2002 score correlated directly with age while SGA did not. Both NRS-2002 and SGA negatively correlated with anthropometric data, ADL, IADL and MMSE. Significant positive correlations were found between nutritional scales and VES-13. For anthropometric data these associations were similar while for ADL, IADL, MMSE and VES-13 were higher for NRS-2002 than for the SGA. These correlations were generally similar in women and men. There were no relationship between GDS and nutritional scales. The results of Pearson’s correlations were very similar.

Table 5 presents the comparison of well-nourished subjects and patients with suggested problems with nutrition according to NRS-2002 (0-2 vs. 3-7) and between the group without problems with nutrition (SGA A) and the group of subjects suspected of malnutrition or malnourished (SGA B + C). Concerning age, nutritional-different subgroups were better discriminated by NRS-2002. Both nutritional scales at given cut-off points similarly discriminated anthropometric data and other CGA tools in the populations of well-nourished vs. malnourished hospitalized older subjects. The sensitivity of NRS-2002 to detect malnutrition was 77.4% and specificity was 87.7% as compared to SGA.

## 4. Discussion

This report is one of the first studies comparing NRS-2002 and SGA with tools from CGA. Both nutritional approaches are widely used in screening and assessment of malnutrition [13]. CGA is a multidisciplinary set of procedures that identifies medical, psychosocial, and functional capabilities of an older adult. CGA is a standard assessment methodology at geriatric wards. Our data indicates that both short nutrition assessment tools are similarly but moderately related to physical and mental function of hospitalized older adults.

There is a variety of tests for screening and assessment of malnutrition like Mini Nutritional Assessment (MNA), Malnutrition Universal Screening Tool (MUST), NRS-2002 and SGA [13,21]. Nevertheless, there is no single tool that can be considered as the universal gold standard for the diagnosis of nutritional status in hospitalized patients [22,23]. SGA and NRS-2002 are among the most widely validated and recommended for older patients [13]. Several studies proved the usefulness of those tools to predict the length of hospital stay or clinical outcome [24,25]. In 124 critically ill patients the SGA rating correlated significantly with percentage of body mass loss, serum albumin level, health status scores and mortality [26]. Malnutrition assessed with SGA in 66 consecutive patients prior to peripheral blood stem cell transplantation was associated with increased length of hospital stay [27]. Both SGA and MNA predicted 3-year mortality in 83 consecutive acute geriatric patients [28]. NRS-2002 and Mini Nutrition Assessment-Short Form (MNA-SF) had similar performance to predict unfavourable clinical outcomes in 705 patients admitted to a Brazilian public university hospital [29]. NRS-2002 was a valuable prognostic tool in 750 adults admitted to the emergency service [30]. In a large multicentre prospective cohort study NRS-2002 was an independent predictor of poor clinical outcome in 5051 patients [31]. In a prospective analysis of 536 hospitalized Chinese patients both NRS-2002 and MNA scores could predict mortality [32].

Several studies compared malnutrition short assessment tools, some of those studies used SGA as a reference method or a “gold standard” [25,33,34,35]. Both MUST and NRS-2002 showed good agreement with SGA in identification of nutritional risk in 577 adult patients admitted to a public emergency room [34]. Comparison of four short nutrition assessment tools (NRS-2002, MUST, SGA and MNA) in 400 patients admitted to the hospital revealed significant differences between the four nutritional assessment tools. The best agreement between the tools was for NRS-2002 with SGA and MUST with SGA. The authors concluded that at admission, NRS-2002 and MUST should be used to screen for nutritional status [36]. On the other hand, in 995 patients assessed at hospital admission NRS-2002 had higher sensitivity and specificity than MUST and Nutritional Risk Index (NRI), as compared to SGA [25]. The sensitivity was 62% and specificity was 93% with the NRS-2002 [25]. The criterion validity of the Malnutrition Screening Tool (MST), MUST, NRS-2002, MNA-SF, modified MST (MST combined with low BMI), and BMI as independent tools was assessed in 693 patients from Vietnam using SGA or low BMI (<18.5 kg/m2) as the reference method. Based on specificity and sensitivity, the first choice for the most appropriate assessment tool for use was the NRS-2002 [37]. Zhang et al. compared SGA and NRS-2002 in 312 oncologic patients [38]. The SGA-A had a higher sensitivity (93.73%) but a poorer specificity (2.30%) than the NRS-2002 <3 points (69.30% and 25.00%, respectively) after comparison with albumin. A high similarity between the SGA and NRS-2002 for evaluating nutritional status was found [38,39]. A systematic review including 111 studies representing 52,911 participants showed that NRS-2002 and SGA had a significant correlation with BMI and several biomarkers of malnutrition. Those results were similar for SGA and NRS-2002 [40]. On the other hand, Ozkalkanli et al. compared NRS-2002 and SGA in predicting the development of complications in patients undergoing orthopaedic surgery. Sensitivity was 50% with the SGA and 69% with the NRS-2002; specificity was 77% with the SGA and 80% with the NRS-2002. The authors concluded that NRS-2002 predicted the development of complications better than the SGA [41].

In the present study, the agreement between the two short nutrition assessment tools was very high. The sensitivity of NRS-2002 to detect malnutrition was 77.4% and specificity was 87.7% as compared to SGA. Interestingly, though several studies linked clinical outcome measures to malnutrition assessment tools and compared different tools, very few studies assessed malnutrition measures in relation to the CGA measurements in older subjects. In one available study the prevalence of malnutrition was 53.6% according to the SGA and 44.6% according to the NRS-2002 in 815 hospitalized patients with an average age of 62.2 years [39]. The prevalence of malnutrition was strongly correlated with the severity of depression and dementia [39]. In another study an important correlation was found between SGA and several cognitive/functional geriatric tests in 81 elderly dialysis patients [42].

In our study, the prevalence of malnutrition was 22% according to NRS-2002 and 15% according to SGA. Both NRS-2002 and SGA showed correlation with anthropometric data and CGA measurements concerning physical and cognitive functioning. The fact that the distribution of both NRS-2002 and SGA was very similar in women and men, and correlations of NRS-2002 and SGA with age, anthropometric data and other CGA tools were generally similar in both sexes provides important practical information about usefulness of both nutritional tools equally in older women and men. Significant association of both tools was also observed with VES-13. VES-13 was used as a measure of frailty, as it is one of the most commonly used instruments [43] with a high sensitivity for predicting the occurrence of disability, mortality and institutionalization [44]. Lack of correlation with age and weaker correlations with physical, cognitive and frailty data for SGA may suggest that NRS-2002 might be more suitable for hospitalized older adults. This potential disparity should be corroborated in future prospective studies. Especially, given recently demonstrated high sensitivity of NRS-2002 for identifying nutritional risk and predictive validity for prolonged hospitalization in older adults with COVID-19 [45]. Adding those physical, cognitive and frailty data to phenotypic and etiologic criteria of malnutrition proposed by the Global Leadership Initiative on Malnutrition (GLIM) might also have enriched diagnosis and severity grading of malnutrition [13].

While this study shows several advantages it also has some limitations. The study was conducted in the “real world” geriatric hospitalized population–in patients with multiple medical problems but being able to respond and perform basic geriatric tests. Therefore, the group of patients was relatively heterogenic and many patients were excluded due to the terminal status or incapacity to perform all tests. Relationship of nutritional status to functional correlates may also be different during long-term hospitalization or in an institutional environment [46]. Secondly, we used only two short nutritional assessment tests–NRS-2002 and SGA. Other nutritional assessment tools like MNA or MUST might have performed better, but they are more difficult to apply in everyday screening practice. Finally, an important aspect of prevention of malnutrition is not only checking the state of nutrition on admission, but also monitoring the nutritional status and its predictive value during and after the hospitalization. Future prospective studies are needed to assess the best and feasible short assessment procedure to predict future outcomes in hospitalized older subjects.

## 5. Conclusions

We can recommend using both NRS-2002 and SGA to detect malnutrition or risk of malnutrition in a routine clinical practice of the geriatric department ward. These tests similarly discriminate the two groups of well-nourished vs. malnourished/at risk older hospitalized patients. Nevertheless, the relationship of both tests to other measures of routine geriatric assessment is moderate and future research should search for further optimisation of nutritional assessment in a geriatric hospital setting.

## Figures and Tables

**Table 1:** Characteristics of the patients—summary statistics for age, anthropometric measurements, ADL, IADL, MMSE, GDS, VES-13, NRS-2002 and SGA.

	All	Women	Men
	(*n* = 622)	(*n* = 431)	(*n* = 191)
Age	81.7 ± 7.78	81.9 ± 7.70	81.4 ± 7.97
Body mass (kg)	65.9 ± 15.5	62.3 ± 14.2	74.3 ± 15.3 ***
Waist circumference (cm)	93.1 ± 13.8	91.1 ± 13.3	97.5 ± 13.8 ***
Calf circumference (cm)	34.6 ± 5.98	34.1 ± 5.78	35.6 ± 6.29 **
Arm circumference (cm)	27.4 ± 4.83	27.1 ± 4.90	28.1 ± 4.63 *
BMI (kg/m^2^)	25.6 ± 4.96	25.5 ± 5.14	25.7 ± 4.55
ADL	4.73 ± 1.77	4.64 ± 1.83	4.94 ± 1.62 *
IADL	5.04 ± 2.86	4.89 ± 2.90	5.38 ± 2.76 *
MMSE	21.6 ± 7.88	21.25 ± 8.00	22.3 ± 7.56
GDS	5.07 ± 3.57	5.27 ± 3.59	4.62 ± 3.51 *
VES-13	6.54 ± 2.92	6.75 ± 2.87	6.06 ± 2.96 **
NRS-2002	1.61 ± 1.25	1.61 ± 1.21	1.62 ± 1.33
SGA	1.16 ± 0.42	1.17 ± 0.42	1.15 ± 0.41

* *p* < 0.05, ** *p* < 0.01, *** *p* < 0.001. BMI, Body Mass Index; ADL, Activities of Daily Living; IADL, Instrumental Activities of Daily Living; MMSE, Mini-Mental State Examination; GDS, Geriatric Depression Scale; VES-13, Vulnerable Elders Survey-13; NRS-2002, Nutrition Risk Screening 2002; SGA, Subjective Global Assessment Form.

**Table 2:** NRS-2002 distribution.

NRS-2002	All (*n* = 622)		Women (*n* = 431)		Men (*n* = 191)	
0	52	8.4%	35	8.1%	17	8.9%
1	363	58.4%	248	57.5%	115	60.2%
2	70	11.3%	55	12.8%	15	7.9%
3	83	13.3%	56	13%	27	14.1%
4	30	4.8%	23	5.3%	7	3.7%
5	15	2.4%	10	2.3%	5	2.6%
6	8	1.3%	3	0.7%	5	2.6%
7	1	0.2%	1	0.2%	0	0%

**Table 3:** SGA distribution.

SGA	All (*n* = 622)		Women (*n* =431)		Men (*n* = 191)	
A	529	85%	363	84.2%	166	86.90%
B	82	13.2%	61	14.2%	21	11%
C	11	1.8%	7	1.6%	4	2.10%

**Table 4:** Spearman correlation coefficients of NRS-2002 and SGA with age, anthropometric data and other Comprehensive Geriatric Assessment measurements.

	All		Women		Men	
	NRS-2002	SGA	NRS-2002	SGA	NRS-2002	SGA
Age	0.30 *	0.03	0.29 *	0.03	0.33 *	0.04
Body mass (kg)	−0.34 *	−0.40 *	−0.37 *	−0.45 *	−0.32 *	−0.33 *
Waist circumference (cm)	−0.32 *	−0.38 *	−0.34 *	−0.43 *	−0.27 *	−0.30 *
Calf circumference (cm)	−0.26 *	−0.34 *	−0.26 *	−0.36 *	−0.25 *	−0.32 *
Arm circumference (cm)	−0.30 *	−0.38 *	−0.31 *	−0.37 *	−0.27 *	−0.39 *
BMI	−0.38 *	−0.43 *	−0.40 *	−0.46 *	−0.33 *	−0.36 *
ADL	−0.28 *	−0.19 *	−0.31 *	−0.20 *	−0.22 *	−0.14
IADL	−0.28 *	−0.14 *	−0.29 *	−0.16 *	−0.24 *	−0.10
MMSE	−0.26 *	−0.13 *	−0.28 *	−0.15 *	−0.21 *	−0.10
GDS	0.06	0.07	0.05	0.04	0.06	0.12
VES-13	0.26 *	0.11 *	0.25 *	0.10 *	0.27 *	0.13

* *p* < 0.05.

**Table 5:** Comparison of the subjects with different nutritional status according to NRS-2002 (NRS 0+1+2 vs. NRS 3-7) and SGA (SGA A vs. SGA B + C).

	NRS 0+1+2 (*n* = 485)	NRS 3-7 (*n* = 137)	SGA A (*n* = 529)	SGA B + C (*n* = 93)
Age	81.2 ± 8.03	83.6 ± 6.51 **	81.6 ± 7.90	82.5 ± 7.03
Men (%)	30.3%	32.1%	31.4%	26.9%
Body mass (kg)	68.1 ± 15.3	58.3 ± 14.2 ***	68.4 ± 15.04	52.03 ± 10.1 ***
Waist circumference (cm)	94.97 ± 13.2	85.7 ± 13.5 ***	95.3 ± 12.98	80.4 ± 11.3 ***
Calf circumference (cm)	35.3 ± 5.82	32.02 ± 5.87 **	35.4 ± 5.87	30.3 ± 4.58 ***
Arm circumference (cm)	27.8 ± 4.69	25.7 ± 5.11 ***	28.1 ± 4.69	23.5 ± 3.67 ***
BMI (kg/m^2^)	26.4 ± 4.80	22.6 ± 4.44 ***	26.4 ± 4.76	20.8 ± 3.11 ***
ADL	4.96 ± 1.59	3.90 ± 2.11 ***	4.88 ± 1.67	3.92 ± 2.09 ***
IADL	5.37 ± 2.73	3.82 ± 3.004 ***	5.22 ± 2.78	4.01 ± 3.14 ***
MMSE	22.3 ± 7.44	19.1 ± 8.901 ***	22.02 ± 7.56	18.9 ± 9.11 ***
GDS	4.99 ± 3.48	5.37 ± 3.89	4.96 ± 3.50	5.73 ± 3.92
VES-13	6.31 ± 2.97	7.35 ± 2.55 ***	6.41 ± 2.93	7.26 ± 2.74 **
SGA	1.04 ± 0.20	1.61 ± 0.63 ***	-	-
NRS-2002	-	-	1.28 ± 0.89	3.45 ± 1.37 ***

** *p* < 0.01; *** *p* < 0.001.

## Data Availability

Not applicable.
